# Evaluation of physical activity before and after respiratory rehabilitation in normal weight individuals with asthma: a feasibility study

**DOI:** 10.3389/fspor.2024.1372048

**Published:** 2024-05-09

**Authors:** Federico Mattia Oliva, Matteo Tarasconi, Alberto Malovini, Martina Zappa, Dina Visca, Elisabetta Zampogna

**Affiliations:** ^1^ Department of Anesthesia and Intensive Care, IRCCS San Raffaele Scientific Institute, Milan, Italy; ^2^ Division of Pulmonary Rehabilitation, Istituti Clinici Scientifici Maugeri IRCCS, Tradate, Italy; ^3^ Laboratory of Informatics and Systems Engineering for Clinical Research, Istituti Clinici Scientifici Maugeri IRCCS, Pavia, Italy; ^4^ Department of Medicine and Surgery, University of Insubria, Varese, Italy

**Keywords:** exercise, rehabilitation, physical activity, sedentary behavior, asthma

## Abstract

**Background:**

Individuals with asthma spend less time engaging in physical activity compared to the general population. Increasing physical activity has become a patient-centered goal for the treatment of treatable traits of individuals with asthma. There are data showing the possible effects of a pulmonary rehabilitation program on physical activity in obese individuals with asthma but not in normal-weight asthmatics. The objective of this feasibility study is to estimate the number of daily steps and time spent on activity in normal-weight individuals with asthma, measured before and after a pulmonary rehabilitation program.

**Methods:**

Normal-weight individuals with moderate to severe asthma were evaluated. The individuals measured their daily steps with an accelerometer for 5 days before and after a pulmonary rehabilitation program. The study was registered on ClinicalTrials.gov: NCT05486689.

**Results:**

In total, 17 participants were enrolled; one dropout and data on the time in activity of two individuals are missing due to a software error during the download. Data from 16 patients were analyzed. The median number of steps/day at baseline was 5,578 (25th, 75th percentiles = 4,874, 9,685) while the median activity time was 214 min (25th, 75th percentiles = 165, 239). After the rehabilitation program, the number of daily steps increased by a median value of 472 (*p*-value = 0.561) and the time in activity reduced by 17 min (*p*-value = 0.357). We also found a significant difference in quality of life, muscle strength, and exercise capacity.

**Conclusions:**

The results of this study make it possible to calculate the sample size of future studies whose main outcome is daily steps in normal-weight individuals with asthma. The difficulties encountered in downloading time in activity data do not allow the same for this outcome.

**Clinical Trial Registration:**

ClinicalTrials.gov, identifier NCT05486689.

## Introduction

Asthma is a chronic lung disease, which leads to several adverse outcomes, including possible lower levels of physical activity (PA) (1). A systematic review showed that adults with asthma had lower levels of total, moderate, and vigorous PA than those without asthma (2). Physical inactivity is an important risk factor; therefore, increasing PA had become a patient-centered goal for the treatment of treatable traits of individuals with asthma (3, 4).

In individuals with asthma, PA is influenced by age, sex, decreased disease control, increased severity as well as co-morbidities such as obesity (5–8).

A recent study examined whether PA was related to body mass index (BMI) and asthma control in 206 asthma patients (9). The results showed that obese individuals (BMI ≥ 30 kg/m^2^) with uncontrolled asthma (Asthma Control Questionnaire score > 0.75) walked approximately 2,000 fewer steps than non-obese individuals with good disease control (*p* ≤ 0.05). PA is also inversely associated with body weight in other populations, as shown by the CARDIA study, a 10-year longitudinal study involving young, bi-racial, and free-living adults (10).

Severe and obese asthmatic individuals have been the subject of several studies that had as their outcome the improvement of PA through the application of exercise programs, pulmonary rehabilitation (PR), and diet. Ma et al. conducted a study of a cohort of obese patients with asthma who were assigned to a 12-month lifestyle intervention aimed at modest weight loss (achieving and maintaining a weight loss of 7% of baseline body weight) and increased PA (achieving and maintaining a minimum of 150 min per week of moderate-intensity PA). The intervention group was compared to a control group that received usual care. The authors concluded that moderately and severely obese adults with uncontrolled asthma showed modest average weight reduction and improvements in PA (11). Freitas et al. confirmed that a comprehensive PR program, including a weight loss program and exercise training, improves PA in moderately to severely obese adults with asthma (12). The results of a recent systematic review revealed some evidence supporting the effectiveness of different supervised or unsupervised interventions, such as walking, running, cycling, low-intensity exercise (i.e., yoga), improving PA in adults with severe asthma (13).

The population of normal-weight asthmatics is also most likely at risk of being inactive but is, to the best of our knowledge, less studied.

To date, there are no data on daily steps walked by normal-weight asthmatic individuals nor data showing the possible effects of a PR program on PA in these individuals.

For this reason, the main objective of this feasibility study was to estimate the median value (25th, 75th percentiles) as well as the mean value [standard deviation (SD)] of the number of daily steps and time spent on activity in minutes in normal-weight individuals with asthma, measured before and after the PR program. The result will allow the formal calculation of the sample size for planning a larger study to evaluate PA in normal-weight individuals with asthma. Moreover, our secondary objective was to investigate changes in respiratory function, physical capacity, dyspnea, and quality of life after the PR program. It is crucial to emphasize that, given the nature of this feasibility study, these outcomes were gathered not to validate the treatment's effectiveness but solely for exploratory purposes (14).

## Methods

The institutional review board and central ethical committee of the Istituti Clinici Scientifici Maugeri IRCCS, Pavia, Italy, approved the study (No 2652, 29/06/2022). The study was performed according to the Declaration of Helsinki. The study was registered on ClinicalTrials.gov (NCT05486689). Patient consent was obtained and the data were treated confidentially.

### Participants

Individuals with a diagnosis of asthma according to the Global Initiative for Asthma (GINA) guidelines (15), and admitted to Istituti Clinici Scientifici Maugeri of Tradate to complete a comprehensive PR program, were screened to assess eligibility.

#### Inclusion criteria

The inclusion criteria were as follows: age ≥50 to ≤75 years, diagnosis of asthma according to GINA steps 3–5, BMI 18.5–24.9 kg/m^2^, and under optimal medical treatment for at least 3 months (15). The age range was determined by identifying the most referenced age group in PR to mitigate potential age-related variability, especially considering the limited sample size.

#### Exclusion criteria

The exclusion criteria were as follows: diagnosis of any other chronic pulmonary disease; smokers or ex-smokers with pack/years ≥10; acute exacerbation of asthma in the last 30 days; engagement in exercise training program in the previous 6 months; history of significant oncological, neurological, or cardiovascular diseases; musculoskeletal impairment and/or medical diseases that are likely to compromise mobility and preclude exercise testing and PR; use of a walker when moving; and reduced spontaneous gait speed (10 m walk test <0.8 m/s) (16, 17).

### Intervention

The enrolled participants received a comprehensive PR program offered by a team consisting of chest physicians, nurses, physiotherapists, dieticians, and psychologists. The three weeks of inpatient PR included education, exercise training, and nutritional and psychological counseling.

Education consisted of at least three individualized 20-min sessions run by a chest physician (addressing asthma pathophysiology, medication, symptoms control, action plan), a nurse (addressing inhalation technique), and a physiotherapist (addressing avoidance strategy, exercise training, and maintenance strategies). In addition, a minimum of three 45-min group sessions led by a dietitian (addressing diet and body weight control), a physiotherapist (addressing PA recommendations and benefits), and a psychologist (addressing lifestyle, stress management, depression, anxiety, and relaxation techniques) were provided.

Endurance training consisted of at least 12 daily sessions of 30 min of supervised incremental exercise training according to symptoms (18), using continuous cycling at 50%–70% of the maximal load calculated on the basis of the baseline 6-min walking test (6MWT) according to Hill (19). The workload was increased by 5 W when participants scored their dyspnea and/or leg fatigue <3 on a modified 10-point Borg Scale (20). The workload was unchanged when the Borg score was 4 or 5 and reduced for scores >5.

Strength training consisted of 12 daily sessions of 30 min of peripheral limb muscle strength training. The training consisted of two sets of 8 repetitions during the first week, which increased to 12 from the second week. Dumbbells and ankle braces were used. The initial load was set at 30%–50% of the maximum voluntary isometric strength (MVS). The workload was varied by 0.5/1 kg following the same method as described above (20).

### Measurements

**Figure 1:**
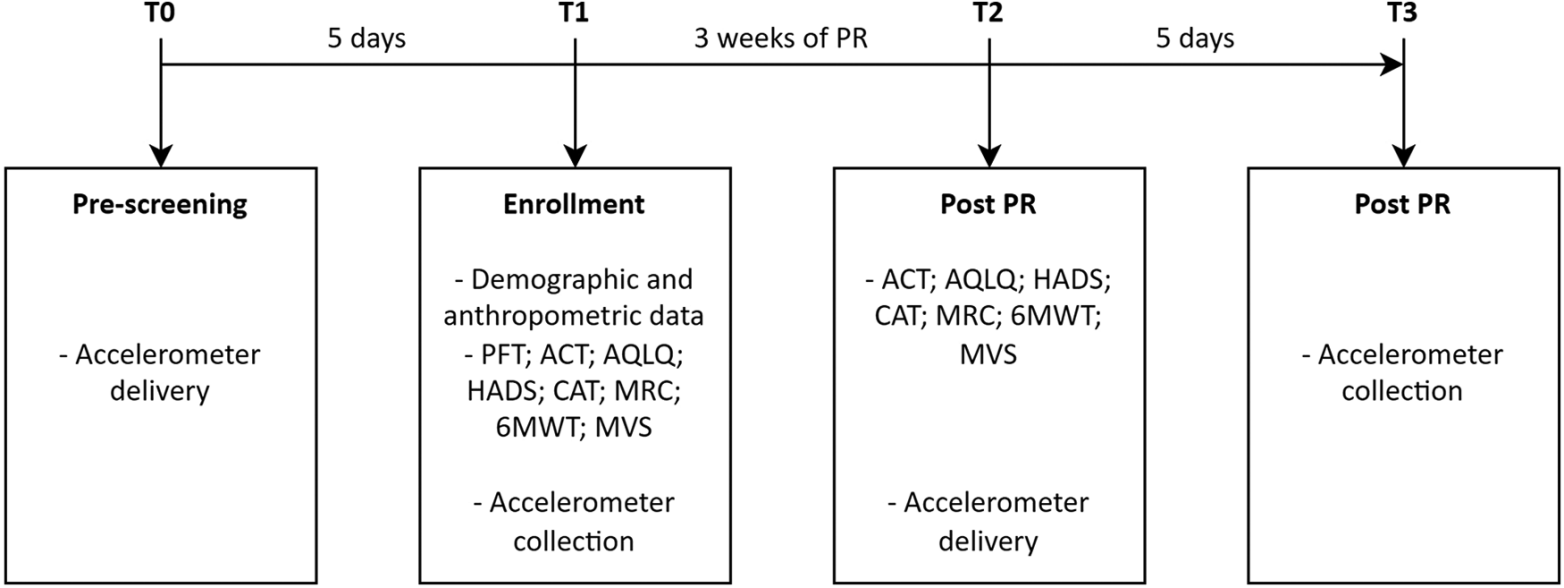
Study timeline. T0–T3, evaluation time; PFT, pulmonary function test; PR, pulmonary rehabilitation.

The study had four time frames as shown in Figure 1. Data about demographics, anthropometrics, relevant controlled treatment according to guidelines (15), co-morbidities with the Cumulative Illness Rating Scale severity and comorbidity (CIRS) (21), dynamic lung volumes according to standards (22) using the predicted values of Quanjer et al. (23), and asthma control with the asthma control test (ACT) were collected (24). Gait speed was assessed using the 10 m walking test that consists of 10 m walk on plain ground at a self-selected speed (16).

PA, measured as the number of daily steps and time spent in activity, was assessed using an accelerometer (Xiaomi® Mi Smart Band 5, China) (25). This device contains an accelerometer and gyroscope, both triaxial, and uses its own algorithm to calculate the number of daily steps and time spent in activity. The data are synchronized via Bluetooth with a specific application (Mi Fit for Android) installed on a smartphone. The data on the number of steps per day were analyzed as reported by the Mi Fit application. Xiaomi® does not disclose the calculation algorithm for time spent in activities. However, it is possible to export from the application a file containing the start and end times of a period of continuous activity detected through the combination of the information provided by the sensors. To calculate the time spent in activity for each day, we combined all the activity intervals provided in this file on a daily basis. The accelerometer was placed on the wrist of the non-dominant arm and maintained 24 h a day for 4 consecutive days excluding the weekend. Upon receiving the accelerometer (T0 and T2), participants were instructed to perform their usual PA (T0 and T2). Returning the accelerometer and checking the data were done after 5 days from delivery (T1 and T3). Data were considered usable if recorded for a minimum of 4 days with a wearing time of ≥8 h during waking hours (26). The individual was classified as sedentary (<5,000 steps/day), low/somewhat active (5,000–9,999 steps/day), and active (≥10,000 steps/day) (27). A 500-step increment was considered clinically significant (28).

Weight and height were measured in all the participants while they were fasting in the morning, wearing light clothing, and without shoes. Body weight and height (in cm to the nearest 0.1 cm) were measured with the same instrument using a measuring slide and a heel plate (Body Weight, BWS-XB, Forlì, Italy).

For lung function, vital capacity (VC), forced expiratory volume in the first second (FEV1), total lung capacity (TLC), and residual volume (RV) were measured by means of a flow-sensing spirometer and a body plethysmograph connected to a computer for data analysis (Masterlab, Jaeger, Wurzburg, Germany). Spirometry tests were performed in accordance with the 2019 ATS/ERS statement, with a minimum of three measurements per test to guarantee the reproducibility of the data (29).

Quality of life was assessed using the self-completed Asthma Quality of Life Questionnaire (AQLQ) (30). The score is in the range of 1–7, with higher scores indicating a better quality of life. A change of 0.5 was considered the minimal clinically important difference (MCID) (31).

Health status was assessed using the COPD assessment test (CAT) (32), assessing globally the impact of cough, sputum, dyspnea, and chest tightness on health status; the score is in the range of 0–40, where a higher score denotes a more severe impact. A change of −2 points was considered the MCID (33).

The severity of dyspnea was evaluated using the Medical Research Council (MRC) scale, which is in the range of 0–4, with higher scores indicating a more severe impact (34). A change of −1 point was usually considered clinically relevant (35).

Exercise capacity was evaluated using the 6MWT according to accepted standards (36) using the predicted values by Enright and Sherrill (37). At the beginning and end of walking, subjective sensations of both dyspnea and leg fatigue were assessed by means of the modified Borg scale (20). A change of 27 m was considered the MCID (38).

Quadriceps and biceps strength was assessed using the MVS (39). The measures were performed with a hand-held dynamometer (KFORCE, SAS Kinvent Biomecanique, Montpellier, France). The body positions for the tests were standardized. To assess the quadriceps, the dynamometer was placed at the lower third of the tibia, perpendicular to the leg and parallel to the floor, and participants were seated with a knee joint angle of 90° and a hip extension of 120° with their arms crossed over the chest. To evaluate the biceps, the dynamometer was positioned at the wrist, to be parallel to the forearm and to the floor. Participants were seated with a shoulder flexion of 0° and an elbow flexion of 90°. Participants performed three MVSs bilaterally, each lasting 5 s, with 30 s of rest. The mean value of the two best tests was recorded (40). Predictive values were calculated using the method proposed by Andrews et al. (41). A change of 10.5% was considered the quadriceps MCID (42, 43) and a change of 20% was considered the biceps MCID (44).

Mood disorder was assessed using the Hospital Anxiety and Depression Scale (HADS), a 14-item scale with seven items each for anxiety and depression subscales. The total score is in the range of 0–21 and a value >8 points denotes anxiety or depression. The MCID was considered −1.3 for HADS-anxiety and −1.4 for HADS-depression (45).

The primary outcome was the number of daily steps and time spent on activity before and after PR.

The secondary outcomes were health-related quality of life (AQLQ), health status (CAT), dyspnea (MRC), exercise capacity (6MWT), quadriceps and biceps strength (MVS), and mood (HADS) before and after PR.

### Statistical methods

Quantitative variable distributions were described in terms of mean ± SD and median (25th, 75th percentiles). Categorical variable distributions were described in terms of absolute and relative (%) frequencies. Change values were computed as the difference between values at T2 and values at T1 (T2 − T1). The two-sided Wilcoxon signed-rank test was applied to test the null hypothesis of no change in terms of quantitative variables distribution between T1 and T2. Non-parametric statistical tests were applied due to the limited size of the analyzed sample. *Post hoc* empirical power calculations have been performed as follows. Individual-level change values (T2 − T1) corresponding to each variable have been simulated 10,000 times imposing sample size, mean and SD deriving from data, and a two-sided one sample Wilcoxon signed-rank test was applied each time to test the null hypothesis of no change between T1 and T2. The statistical power has been then estimated as the proportion of tests reaching *p*<0.05 over the number of simulations performed. Quantitative changes were dichotomized according to the corresponding MCID values. Statistical analyses were performed using the R software environment for statistical computing and graphics version 4.2.2 (https://www.r-project.org/). A *p*-value <0.05 was considered statistically significant.

### Sample size determination

The determination of the sample size was guided by Julius’ study published in 2005, which recommends a minimum sample size of 12 individuals per group (46). Given a single group, we chose to increase the sample size to 15 participants and factored in a 10% addition to allow for potential dropouts, resulting in a total of 17 participants.

## Results

**Figure 2:**
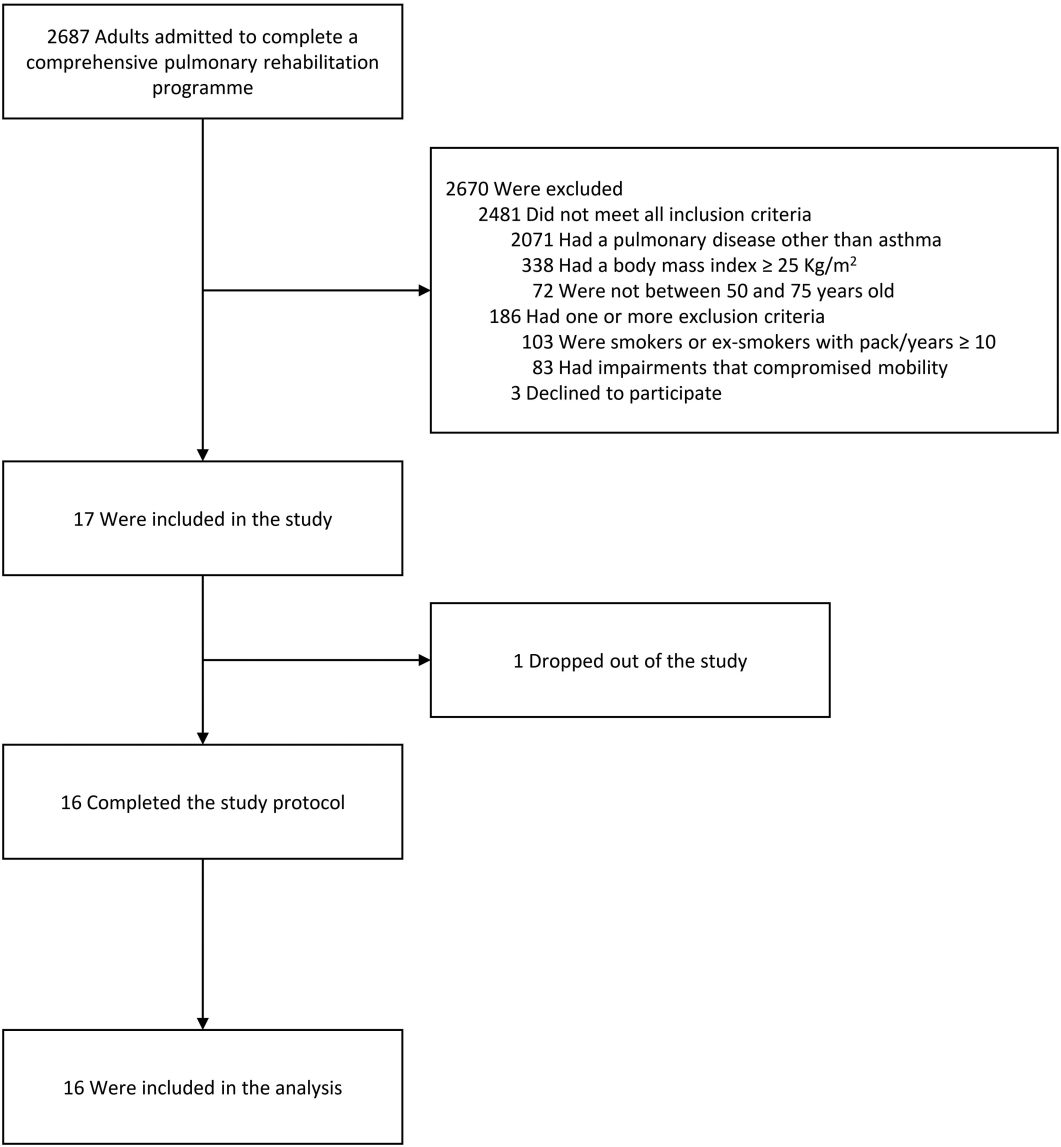
Flow chart of the study.

In total, 17 individuals were enrolled between July 2022 and May 2023. The flow chart of the study is shown in Figure 2. One individual discontinued the study due to an adverse event that occurred in the screening phase (community acquired pneumonia). All other participants concluded the study with a participation rate of more than 94%. Of these 16 participants, two were unable to perform spirometry due to technical issues, and for two others, data on time spent in activity were missing due to a download error. Data from 16 patients were analyzed.

The demographic, anthropometric, physiological, and clinical characteristics of the enrolled patients at T1 are reported in Table 1. The participants were mostly adult women with not well controlled asthma in GINA step 5, low bronchial obstruction, and a median of two co-morbidities. All participants completed 12–14 training sessions during the PR program.

**Table 1:** Characteristics of individuals at baseline.

	Distribution	*N*
Male, *n* (%)	4 (25%)	16
Age, y.o.	62 (57, 68)	16
	61.56 ± 7.68	
CIRS severity score	1.4 (1.3, 1.5)	16
	1.43 ± 0.22	
CIRS comorbidity score	2 (1, 3)	16
	2.44 ± 1.59	
Asthma GINA steps, *n* (%) 3: Low-dose ICS-formoterol 4: Medium-dose maintenance ICS-formoterol 5: Add-on LAMA, high-dose ICS-formoterol	1 (6.25%) 4 (25.00%) 11 (68.75%)	16
ACT score	20 (16, 22)	16
	18.50 ± 4.63	
Ex-smokers, *n* (%)	3 (18.75%)	16
BMI, kg/m^2^	22.75 (22.37, 23.35)	16
	22.92 ± 1.22	
FEV1, L	2.06 (1.85, 2.36)	16
	2.12 ± 0.50	
FEV1, % predicted	81 (69.50, 100.75)	16
	84.19 ± 19.84	
FVC, L	2.83 (2.63, 3.17)	16
	3.06 ± 0.73	
FVC, % predicted	96 (86.75, 104.25)	16
	95.12 ± 15.58	
FEV1/FVC %	68.93 (66.15, 77.92)	16
	69.82 ± 11.23	

*N*, number of observations; y.o., years old; ICS, inhaled corticosteroids; LAMA, long acting muscarinic agonists; FVC, forced vital capacity.

Data are presented as absolute (relative frequency, %), median (25th, 75th percentiles), and mean ± SD.

All the participants used the accelerometer for all the time requested, without reporting any fit problems. As shown in Table 2, the median number of daily steps recorded at T1 and T2 were 5,578 and 6,979, respectively (*p* = 0.562). At T1, 5 (31%) participants were classified as sedentary, 8 (50%) as low/somewhat active, and 3 (19%) as active. The median time spent in activity at T1 and T2 was 214 and 174 min, respectively (*p* = 0.358). At T2, a statistically significant improvement of AQLQ, CAT, MRC, 6MWT, HADS, and quadriceps MVS was recorded (*p*<0.05).

**Table 2:** Differences between pre to post pulmonary rehabilitation.

	T1	T2	*p*-value
Steps, *n* (*N* = 16)	5,578 (4,874, 9,685)	6,979 (5,098, 10,070)	0.562
	7,246 ± 3,248	8,040 ± 4,047	
Time in activity, min (*N* = 14)	214 (165, 239)	174 (163, 227)	0.358
	207 ± 69	205 ± 76	
AQLQ score (*N* = 16)	5.17 (3.85, 6.10)	5.62 (4.90, 6.36)	0.049*
	5.02 ± 1.28	5.47 ± 1.22	
HADS-depression score (*N* = 16)	4.5 (3.75, 8)	2 (2, 4.25)	0.025*
	5.38 ± 2.87	3.38 ± 2.87	
HADS-anxiety score (*N* = 16)	4.50 (3.00, 7.00)	2.00 (1.75, 6.00)	0.029*
	5.12 ± 2.87	3.44 ± 2.66	
CAT score (*N* = 16)	13.5 (9.0, 21.5)	3.5 (3.0, 9.0)	0.001*
	14.25 ± 8.53	6.00 ± 5.13	
MRC score (*N* = 16)	1 (1, 2)	0 (0, 1)	0.005*
	1.12 ± 0.72	0.50 ± 0.63	
MVS bic, kg (*N* = 16)	12.05 (9.62, 14.57)	14.55 (11.97, 17.35)	0.252
	13.17 ± 4.87	14.51 ± 4.38	
MVS bic, % predicted (*N* = 16)	67.00 (53.75, 89.00)	78.50 (65.75, 91.75)	0.326
	70.06 ± 19.33	77.12 ± 17.09	
MVS quad, kg (*N* = 16)	17.50 (14.10, 23.45)	25.45 (18.65, 29.92)	0.003*
	18.22 ± 5.35	25.96 ± 10.13	
MVS quad, % predicted (*N* = 16)	57.50 (46.25, 69.00)	77.50 (66.25, 95.75)	0.004*
	60.62 ± 19.01	83.81 ± 25.70	
6MWT, m (*N* = 16)	538 (504, 558)	561 (524, 590)	0.002*
	528 ± 55	563 ± 63	
6MWT, % predicted (*N* = 16)	103 (93, 108)	108 (95, 118)	0.003*
	101 ± 15	108 ± 17	

*N*, number of observations; bic, biceps; quad, quadriceps.

Data are presented as median (25th, 75th percentiles) and mean ± SD.

* *p*-value <0.05.

*Post hoc* empirical power calculations, based on sample sizes and data-derived estimates, showed that the statistical power to detect significant changes between T1 and T2 exceeded 85% for CAT and MRC scores, quadriceps MVS (in kg and % predicted), and 6MWT (in m and % predicted). For AQLQ scores, HADS-depression, and HADS-anxiety, the power was in the range of 46%–63%. However, it was below 26% for daily steps, time spent in activity, and biceps MVS (in kg and % predicted).

Table 3 describes the change of outcome measures and the percentage of participants reaching the MCID.

**Table 3:** Pre to post-secondary outcomes changes and number (%) of subjects reaching MCID.

	Change	MCID
AQLQ score	0.33 (−0.09, 0.79)	7 (43.75)
	0.46 ± 0.88	
HADS-depression score	−2.00 (−4.00, 1.00)	9 (56.25)
	−2.00 ± 3.08	
HADS-anxiety score	−1.00 (−3.00, −0.75)	7 (43.75)
	−1.69 ± 2.63	
CAT score	−7.00 (−10.50, −3.75)	14 (87.5)
	−8.25 ± 6.40	
MRC score	−1 (−1, 0)	9 (56.25)
	−0.62 ± 0.62	
MVS bic, %	5.88 (−9.89, 32.55)	5 (31.25)
	16.20 ± 37.11	
MVS quad, %	29.47 (7.22, 99.35)	11 (68.75)
	48.70 ± 56.80	
6MWT, m	35.50 (27.75, 48.00)	13 (81.25)
	35.50 ± 27.52	

bic, biceps; quad, quadriceps.

Data are presented as absolute (relative frequency, %), median (IQR), and mean ± SD. Change values were computed as value at T2 − value at T1 except for MVS bic. (%) and MVS quad. (%) that were computed as [(value at T2 − value at T1)/value at T1] × 100.

## Discussion

This feasibility study allowed us to provide an estimate of the number of daily steps and time spent in activity in normal-weight individuals with asthma, before and after PR. This information will be necessary to calculate the sample size of a larger study focusing on PA on daily life on the same population in the future.

During the baseline evaluation, it was observed that most enrolled individuals were sedentary or somewhat active. Only three individuals were found to walk almost 10,000 steps per day, which is considered a standard cut point for good health status (47). A systematic review by Cordova-Rivera et al. found that people with asthma do less PA than people without this condition and showed that the level of activity in asthma seemed to be influenced by age, sex, and disease severity (1). The authors found that people with asthma walked an average of 8,390 steps day, while the severe asthma subgroup reached 5,800 steps/day. These data were similar to our results, where the sample was mainly composed of individuals with severe asthma.

Body weight could also affect PA. A recent paper by Rockette-Wagner et al. studied the relationship between PA and BMI in 260 adults with asthma (9). The data showed that the average daily steps were significantly related to BMI (*p* < 0.001), and this association was attenuated and not significant after adjusting for covariate. However, even after adjustment each 1-unit increase in BMI was associated with a small significant decrease in average minutes/day of moderate to vigorous activity (−2.2; 95% confidence interval −3.6 to −0.3; *p* = 0.03). The improvement in the number of steps is lower in normal-weight individuals with asthma compared to overweight or obese severe people with asthma, as reported by McLoughlin et al. (13).

After completing the rehabilitation program, even though there was an improvement of daily steps, this change was not statistically significant. Furthermore, the variation should be considered clinically important according to Banach et al. who evidenced that a 500-step increment was associated with a 7% decrease in cardiovascular mortality (28). On the other hand, our findings suggest a slight reduction in the time spent in activity after PR. This decrease, which is not statistically significant and which contrasts with the increase in the number of daily steps, could be influenced by factors such as our relatively small sample size and challenges with data retrieval, which further diminished our sample. Notably, prior research has produced mixed conclusions on the impact of PR on time spent in activity (48). Furthermore, the absence of a universally accepted and objective definition for this metric, compounded by variations in its calculation across different devices (49), diminishes its suitability for sample size determination. Given these limitations, to ensure objectivity, future studies investigating the effectiveness of a treatment in modifying PA should use the number of steps per day rather than time spent in activity to assess treatment effect and determine sample size.

Promoting an active lifestyle has become increasingly important in recent years for individuals of all ages, both healthy and with chronic diseases. In particular, PA appears to have a beneficial effect on asthmatic individuals from a pathophysiological perspective. Exercise has been observed to have an anti-inflammatory effect, reducing the blood levels of some inflammatory interleukins, improving bronchial hyperreactivity, and reducing the inflammatory state of the lungs. Furthermore, obesity is associated with higher levels of pro-inflammatory molecules, which can lead to an increased airway inflammatory response and worsening of symptoms (50). Therefore, increasing PA in asthmatic individuals may contribute to better weight control, leading to a decrease in the inflammatory state and an improvement in symptoms.

Despite the constant technological advancement, it remains very complex to study the PA level of individuals, which is strongly influenced by numerous factors; environmental, cultural, social, and personal factors could all impact PA (51). Indeed, we observed a very high variability in the number of daily steps (range 3,476–15,816).

During the study's data collection phase, we encountered technical issues when downloading the recorded data. We had to contact tech support twice but some data about time-on-movement for two participants were lost. This issue, as reported above, undoubtedly impacted the results, diminishing their accuracy and reliability. It will be important to consider this factor when planning future studies that aim to investigate PA using accelerometers, especially if the same devices used for this study are planned. One possible solution to this issue is to use advanced devices supported by a dedicated IT support, which would enable remote access to real-time data collection. This configuration would facilitate the prompt identification and resolution of any issues.

Our results seem to also show that PR has a positive effect in improving exercise capacity, muscle strength, health and mood status, and quality of life. These findings are consistent with previous research that has demonstrated the effectiveness of PR programs in enhancing exercise capacity and reducing symptoms of anxiety and depression in individuals with asthma (50, 52). However, it remains unclear whether PR is effective in improving the quality of life of people with asthma, as studies have shown mixed results on this outcome (50, 52). It is important to note that even in our study, the significance level for quality of life is borderline (*p* = 0.049). PR seems to have a possible effect on PA as well; however, for its impact to be significant, it would probably have to be enhanced by specific and tailored interventions. Since individuals with asthma are known to lead more sedentary lives than healthy individuals (2), it is desirable that future studies investigate this aspect. Promoting an active lifestyle through PA enhancement in individuals with asthma may help to reduce the impact of clinical conditions associated with a sedentary lifestyle, such as obesity, anxiety disorders, and depression, which are often linked to asthma and can lead to a reduced quality of life (50).

This study does have some limitations. First, due to the small sample size, we are unable to generalize our findings to the entire asthmatic population. Results from *post hoc* empirical power calculations showed that the present study is underpowered (statistical power <80%) to detect statistically significant changes for some of the analyzed variables given the significance level as well as the sample size and estimates derived from the data. The results from these analyses should therefore be interpreted with caution. Second, the accuracy of the estimates was further diminished by the fact that we lost some data due to technical issues. However, our study had, on the one hand, the objective of estimating the amount of PA in a particular population of asthmatic individuals to obtain data to be used for the design of future, larger studies and, on the other hand, to test the feasibility of this approach for assessing PA.

In conclusion, the results of this study make it possible to calculate the sample size of future studies whose main outcome is daily steps in normal-weight individuals with asthma. The difficulties encountered in downloading time in activity data do not allow the same for this outcome.

## Data Availability

The deidentified data supporting the conclusions of this article will be made available by the authors upon reasonable request.

## References

[1] Cordova-Rivera L Gibson PG Gardiner PA McDonald VM. A systematic review of associations of physical activity and sedentary time with asthma outcomes. J Allergy Clin Immunol Pract. (2018) 6(6):1968–81.e2. 10.1016/j.jaip.2018.02.027 29510231

[2] Xu M Lodge CJ Lowe AJ Dharmage SC Cassim R Tan D Are adults with asthma less physically active? A systematic review and meta-analysis. J Asthma. (2021) 58(11):1426–43. 10.1080/02770903.2020.1810273 32791878

[3] Ram FSF Robinson S Black PN Picot J. Physical training for asthma. Cochrane Database of Sys Rev. (2005) (4):CD001116. 10.1002/14651858.CD001116.pub2 16235280

[4] McDonald VM Gibson PG. Treatable traits in asthma: moving beyond diagnostic labels. Med J Aust. (2022) 216(7):331–3. 10.5694/mja2.51464 35342966 PMC9313553

[5] van ‘t Hul AJ Frouws S van den Akker E van Lummel R Starrenburg-Razenberg A van Bruggen A Decreased physical activity in adults with bronchial asthma. Respir Med. (2016) 114:72–7. 10.1016/j.rmed.2016.03.016 27109814

[6] Bahmer T Waschki B Schatz F Herzmann C Zabel P Kirsten AM Physical activity, airway resistance and small airway dysfunction in severe asthma. Eur Respir J. (2017) 49(1):1601827. 10.1183/13993003.01827-2016 28052957

[7] Bruno A Uasuf CG Insalaco G Barazzoni R Ballacchino A Gjomarkaj M Nutritional status and physical inactivity in moderated asthmatics: a pilot study. Medicine (Baltimore). (2016) 95(31):e4485. 10.1097/MD.0000000000004485 27495092 PMC4979846

[8] Carpagnano GE Sessa F Scioscia G Lacedonia D Foschino MP Venuti MP Physical activity as a new tool to evaluate the response to omalizumab and mepolizumab in severe asthmatic patients: a pilot study. Front Pharmacol. (2020) 10:1630. 10.3389/fphar.2019.01630 32038267 PMC6992710

[9] Rockette-Wagner B Wisnivesky JP Holguin F Ankam J Arora A Federmann E The relationships between physical activity and asthma control and body mass index (BMI) in patients with asthma. J Asthma. (2024) 61(3):194–202. 10.1080/02770903.2023.2260868 37847059

[10] Schmitz K Jacobs D Leon A Schreiner P Sternfeld B. Physical activity and body weight: associations over ten years in the CARDIA study. Int J Obes. (2000) 24(11):1475–87. 10.1038/sj.ijo.0801415 11126345

[11] Ma J Strub P Xiao L Lavori PW Camargo CA Wilson SR Behavioral weight loss and physical activity intervention in obese adults with asthma. A randomized trial. Ann Am Thorac Soc. (2015) 12(1):1–11. 10.1513/AnnalsATS.201406-271OC 25496399 PMC4342805

[12] Freitas PD Silva AG Ferreira PG Da Silva A Salge JM Carvalho-Pinto RM Exercise improves physical activity and comorbidities in obese adults with asthma. Med Sci Sports Exerc. (2018) 50(7):1367–76. 10.1249/MSS.0000000000001574 29432326

[13] McLoughlin RF Clark VL Urroz PD Gibson PG McDonald VM. Increasing physical activity in severe asthma: a systematic review and meta-analysis. Eur Respir J. (2022) 60(6):2200546. 10.1183/13993003.00546-2022 35896208 PMC9753478

[14] El-Kotob R Giangregorio LM. Pilot and feasibility studies in exercise, physical activity, or rehabilitation research. Pilot Feasibility Stud. (2018) 4(1):137. 10.1186/s40814-018-0326-0 30123527 PMC6090705

[15] Global Initiative for Asthma. Global Strategy for Asthma Management and Prevention. (2023). Available online at: https://ginasthma.org/ (accessed November 11, 2023).

[16] Perera S Mody SH Woodman RC Studenski SA. Meaningful change and responsiveness in common physical performance measures in older adults. J Am Geriatr Soc. (2006) 54(5):743–9. 10.1111/j.1532-5415.2006.00701.x 16696738

[17] Negrini F Gasperini G Guanziroli E Vitale JA Banfi G Molteni F. Using an accelerometer-based step counter in post-stroke patients: validation of a low-cost tool. Int J Environ Res Public Health. (2020) 17(9):3177. 10.3390/ijerph17093177 32370210 PMC7246942

[18] Maltais F LeBlanc P Jobin J Bérubé C Bruneau J Carrier L Intensity of training and physiologic adaptation in patients with chronic obstructive pulmonary disease. Am J Respir Crit Care Med. (1997) 155(2):555–61. 10.1164/ajrccm.155.2.9032194 9032194

[19] Hill K Jenkins SC Cecins N Philippe DL Hillman DR Eastwood PR. Estimating maximum work rate during incremental cycle ergometry testing from six-minute walk distance in patients with chronic obstructive pulmonary disease. Arch Phys Med Rehabil. (2008) 89(9):1782–7. 10.1016/j.apmr.2008.01.020 18760164

[20] Borg GA. Psychophysical bases of perceived exertion. Med Sci Sports Exerc. (1982) 14(5):377–81. 7154893

[21] Linn BS Linn MW Gurel L. Cumulative illness rating scale. J Am Geriatr Soc. (1968) 16(5):622–6. 10.1111/j.1532-5415.1968.tb02103.x 5646906

[22] Celli BR MacNee W, ATS/ERS Task Force. Standards for the diagnosis and treatment of patients with COPD: a summary of the ATS/ERS position paper. Eur Respir J. (2004) 23(6):932–46. 10.1183/09031936.04.00014304 15219010

[23] Quanjer PH Stanojevic S Cole TJ Baur X Hall GL Culver BH Multi-ethnic reference values for spirometry for the 3–95-yr age range: the global lung function 2012 equations. Eur Respir J. (2012) 40(6):1324–43. 10.1183/09031936.00080312 22743675 PMC3786581

[24] Juniper EF O′byrne PM Guyatt GH Ferrie PJ King DR. Development and validation of a questionnaire to measure asthma control. Eur Respir J. (1999) 14(4):902. 10.1034/j.1399-3003.1999.14d29.x 10573240

[25] Lugones-Sanchez C Sanchez-Calavera MA Repiso-Gento I Adalia EG Ramirez-Manent JI Agudo-Conde C Effectiveness of an mHealth intervention combining a smartphone app and smart band on body composition in an overweight and obese population: randomized controlled trial (EVIDENT 3 study). JMIR Mhealth Uhealth. (2020) 8(11):e21771. 10.2196/21771 33242020 PMC7728540

[26] Lövström L Emtner M Alving K Nordvall L Borres MP Janson C High levels of physical activity are associated with poorer asthma control in young females but not in males. Respirology. (2016) 21(1):79–87. 10.1111/resp.12671 26581686

[27] Tudor-Locke C Bassett DR. How many steps/day are enough?: preliminary pedometer indices for public health. Sports Med. (2004) 34(1):1–8. 10.2165/00007256-200434010-00001 14715035

[28] Banach M Lewek J Surma S Penson PE Sahebkar A Martin SS The association between daily step count and all-cause and cardiovascular mortality: a meta-analysis. Eur J Prev Cardiol. (2023) 30(18):1975–85. 10.1093/eurjpc/zwad229 37555441

[29] Graham BL Steenbruggen I Miller MR Barjaktarevic IZ Cooper BG Hall GL Standardization of spirometry 2019 update. An Official American Thoracic Society and European Respiratory Society Technical Statement. Am J Respir Crit Care Med. (2019) 200(8):e70–88. 10.1164/rccm.201908-1590ST 31613151 PMC6794117

[30] Juniper EF Buist AS Cox FM Ferrie PJ King DR. Validation of a standardized version of the asthma quality of life questionnaire. Chest. (1999) 115(5):1265–70. 10.1378/chest.115.5.1265 10334138

[31] Juniper EF Guyatt GH Willan A Griffith LE. Determining a minimal important change in a disease-specific quality of life questionnaire. J Clin Epidemiol. (1994) 47(1):81–7. 10.1016/0895-4356(94)90036-1 8283197

[32] Kurashima K Takaku Y Ohta C Takayanagi N Yanagisawa T Sugita Y. COPD assessment test and severity of airflow limitation in patients with asthma, COPD, and asthma—COPD overlap syndrome. Int J Chron Obstruct Pulmon Dis. (2016) 11:479–87. 10.2147/COPD.S97343 27019598 PMC4786066

[33] Kon SSC Canavan JL Jones SE Nolan CM Clark AL Dickson MJ Minimum clinically important difference for the COPD assessment test: a prospective analysis. Lancet Respir Med. (2014) 2(3):195–203. 10.1016/S2213-2600(14)70001-3 24621681

[34] Mahler DA Wells CK. Evaluation of clinical methods for rating dyspnea. Chest. (1988) 93(3):580–6. 10.1378/chest.93.3.580 3342669

[35] de Torres JP Pinto-Plata V Ingenito E Bagley P Gray A Berger R Power of outcome measurements to detect clinically significant changes in pulmonary rehabilitation of patients with COPD. Chest. (2002) 121(4):1092–8. 10.1378/chest.121.4.1092 11948037

[36] Holland AE Spruit MA Troosters T Puhan MA Pepin V Saey D An official European Respiratory Society/American Thoracic Society technical standard: field walking tests in chronic respiratory disease. Eur Respir J. (2014) 44(6):1428–46. 10.1183/09031936.00150314 25359355

[37] Enright PL Sherrill DL. Reference equations for the six-minute walk in healthy adults. Am J Respir Crit Care Med. (1998) 158(5):1384–7. 10.1164/ajrccm.158.5.9710086 9817683

[38] Zampogna E Ambrosino N Centis R Cherubino F Migliori GB Pignatti P Minimal clinically important difference of the 6-min walking test in patients with asthma. Int J Tuberc Lung Dis. (2021) 25(3):215–21. 10.5588/ijtld.20.0928 33688810

[39] Lee SY Jo ME. Comparison of maximum voluntary isometric contraction of the biceps on various posture and respiration conditions for normalization of electromyography data. J Phys Ther Sci. (2016) 28(11):3007–10. 10.1589/jpts.28.3007 27942110 PMC5140790

[40] Nyberg A Saey D Martin M Maltais F. Test-re-test reliability of quadriceps muscle strength measures in people with more severe chronic obstructive pulmonary disease. J Rehabil Med. (2018) 50(8):759–64. 10.2340/16501977-2354 30004108

[41] Andrews AW Thomas MW Bohannon RW. Normative values for isometric muscle force measurements obtained with hand-held dynamometers. Phys Ther. (1996) 76(3):248–59. 10.1093/ptj/76.3.248 8602410

[42] Iwakura M Okura K Kubota M Sugawara K Kawagoshi A Takahashi H Estimation of minimal clinically important difference for quadriceps and inspiratory muscle strength in older outpatients with chronic obstructive pulmonary disease: a prospective cohort study. Phys Ther Res. (2021) 24(1):35–42. 10.1298/ptr.E10049 33981526 PMC8111409

[43] Vaidya T Beaumont M de Bisschop C Bazerque L Le Blanc C Vincent A Determining the minimally important difference in quadriceps strength in individuals with COPD using a fixed dynamometer. Int J Chron Obstruct Pulmon Dis. (2018) 13:2685–93. 10.2147/COPD.S161342 30214186 PMC6124469

[44] Lang CE Edwards DF Birkenmeier RL Dromerick AW. Estimating minimal clinically important differences of upper-extremity measures early after stroke. Arch Phys Med Rehabil. (2008) 89(9):1693–700. 10.1016/j.apmr.2008.02.022 18760153 PMC2819021

[45] Wynne SC Patel S Barker RE Jones SE Walsh JA Kon SS Anxiety and depression in bronchiectasis: response to pulmonary rehabilitation and minimal clinically important difference of the hospital anxiety and depression scale. Chron Respir Dis. (2020) 17:147997312093329. 10.1177/1479973120933292 PMC7301664 32545998

[46] Julious SA. Sample size of 12 per group rule of thumb for a pilot study. Pharm Stat. (2005) 4(4):287–91. 10.1002/pst.185

[47] Office of the Surgeon General (US). Step It Up! the Surgeon General’s Call to Action to Promote Walking and Walkable Communities [Internet]. Washington, DC: US Department of Health and Human Services (2015). Publications and Reports of the Surgeon General. Available online at: http://www.ncbi.nlm.nih.gov/books/NBK538433/ (accessed March 25, 2024). 30860691

[48] Spruit MA Pitta F McAuley E ZuWallack RL Nici L. Pulmonary rehabilitation and physical activity in patients with chronic obstructive pulmonary disease. Am J Respir Crit Care Med. (2015) 192(8):924–33. 10.1164/rccm.201505-0929CI 26161676

[49] Butte NF Ekelund U Westerterp KR. Assessing physical activity using wearable monitors: measures of physical activity. Med Sci Sports Exerc. (2012) 44(1S):S5–12. 10.1249/MSS.0b013e3182399c0e 22157774

[50] De Lima FF Pinheiro DHA Carvalho CRFD. Physical training in adults with asthma: an integrative approach on strategies, mechanisms, and benefits. Front Rehabil Sci. (2023) 4:1115352. 10.3389/fresc.2023.1115352 36873818 PMC9982132

[51] Seefeldt V Malina RM Clark MA. Factors affecting levels of physical activity in adults. Sports Med. (2002) 32(3):143–68. 10.2165/00007256-200232030-00001 11839079

[52] Osadnik CR Gleeson C McDonald VM Holland AE. Pulmonary rehabilitation versus usual care for adults with asthma. Cochrane Database Syst Rev. (2022) (8):CD013485. 10.1002/14651858.CD013485.pub2 35993916 PMC9394585

